# Resting heart rate variability predicts self-reported difficulties in emotion regulation: a focus on different facets of emotion regulation

**DOI:** 10.3389/fpsyg.2015.00261

**Published:** 2015-03-10

**Authors:** DeWayne P. Williams, Claudia Cash, Cameron Rankin, Anthony Bernardi, Julian Koenig, Julian F. Thayer

**Affiliations:** ^1^Department of Psychology, The Ohio State UniversityColumbus, OH, USA; ^2^The Rollins School of Public Health, Emory UniversityAtlanta, GA, USA

**Keywords:** heart rate variability, emotion regulation, rumination, anxiety, impulse control, emotional clarity, inhibition

## Abstract

The *Model of Neurovisceral Integration* suggests that vagally mediated heart rate variability (vmHRV) represents a psychophysiological index of inhibitory control and thus, is associated with emotion regulation capacity. Over the past decade, growing empirical evidence supports this notion, showing that those with higher resting vmHRV can regulate negative emotions more adequately. However, to our knowledge, no study has previously examined how resting vmHRV may relate to everyday perceived difficulties in emotion regulation. The present study attempts to examine such relationship in 183 undergraduate students (98 female, 60 minority, mean Age = 19.34). Resting vmHRV was collected during a 5-min resting baseline period, and everyday difficulties in emotion regulation were assessed using the Difficulties in Emotion Regulation Scale (DERS). Controlling for potential covariates (including both trait anxiety and rumination), results revealed a negative relationship between resting vmHRV and DERS such that lower resting vmHRV was associated with greater difficulties in emotional regulation, especially a lack of emotional clarity and impulse control, as indicated by the respective subscales of the DERS. These findings provide further evidence for the *Neurovisceral Integration Model*, suggesting that emotion regulation and autonomic regulation share neural networks within the brain. Moreover, the present study extends prior research by highlighting two distinct facets of emotion regulation (impulse control and emotional clarity) that should be of particular interest when investigating the link between emotion regulation, resting vmHRV, and related health outcomes including morbidity and mortality.

## Introduction

“To act wisely, we must see clearly.”

(Easwaren, 2010, p. 184).

Emotion regulation (ER) is defined as a process by which individuals modify their emotional experiences, expressions, and subsequent physiological responses (Aldao, 2013). Inhibitory control is a key mechanism for successful ER—individuals are required to inhibit prepotent emotional responses in service of more desirable and appropriate ones (Lane et al., 2009; Thayer et al., 2012). Emotional responses that are consistent with environmental demands represent adaptive ER and promote physical and mental health. In contrast, emotional responses that are inconsistent with environmental demands (e.g., impulsive emotional responses) represent maladaptive ER, and predict disease and mortality (Thayer and Lane, 2000; Thayer et al., 2012). To better understand psychophysiological mechanisms linking inhibition, ER, and overall health, Thayer and Lane (2000) proposed that the characteristic beat-to-beat variability in the heart rate (HR) time series—heart rate variability (HRV)—serves not only as an index of healthy heart function (Thayer and Lane, 2007), but as an readily available index and measure of inhibitory control, and more specifically, ER capacity.

### The neurovisceral integration model (NIM)

Executive brain areas, such as the prefrontal cortex, exert an inhibitory influence on sub-cortical structures, such as the amygdala, allowing the organism to adaptively respond to demands from the environment, and organize their emotional and behavioral responses effectively (Thayer et al., 2012). Thus, at rest, active cortical brain areas are indicative of greater inhibitory and ER (Thayer et al., 2012). Converging evidence suggests that these core sets of neural structures are not only responsible for inhibition, but also the regulation of the autonomic nervous system (ANS) (re)activity (Hansen et al., 2004). The heart (and other peripheral organs) is under tonic inhibitory control by the ANS. More specifically, this influence is characterized by a relative dominance of the parasympathetic nervous system (PNS) over influences of the sympathetic nervous system (SNS) (Thayer and Lane, 2009; Thayer et al., 2012). Vagal parasympathetic control represents the major descending inhibitory pathway (DIP), adaptively regulating physiological functions (i.e., immune, inflammatory, and cardiac function, Thayer and Sternberg, 2006; Weber et al., 2010) shaped by psychological processes such as ER (Thayer and Lane, 2000; Thayer and Sternberg, 2010). Therefore, the NIM posits that vagally mediated HRV (vmHRV) may be more than just a simple index of healthy heart function, but may in fact serve as readily available measure and index of the degree to which the brain's integrative system for adaptive regulation provides flexible control over the periphery. Overall, it is suggested that this common reciprocal inhibitory cortico-subcortical neural circuit serves as the structural link between psychological processes such as ER, and health-related physiological processes, and that this circuit can be indexed by vmHRV (Thayer et al., 2012).

### Emotion regulation and HRV

Adopting the NIM framework, researchers have explored various contexts where individuals with low resting vmHRV fail at and/or have difficulties with ER, in addition to downstream physical and mental consequences (as indexed by phasic or acute changes in vmHRV) (Carrico et al., 2006; Lane et al., 2009; Melzig et al., 2009; Geisler et al., 2010; Aldao et al., 2012). For example, there is evidence suggesting that those with high vmHRV, in comparison to those with lower levels, show appropriate emotional responses, as indexed by emotion-modulated startle responses, fear-potentiated startle responses, phasic heart rate responses, and behavioral emotional responses (Melzig et al., 2009). More recently, research showed that those with higher vmHRV are better able to suppress unwanted thoughts in comparison to individuals with low vmHRV (Gillie et al., 2014). While this substantial amount of evidence supports the link between vmHRV and ER, studies have not yet directly examined how resting vmHRV may predict individuals' perceived difficulties in ER, along with the strength of the association between resting HRV and varying facets of ER.

### Multifaceted conceptualization of emotion regulation

Indeed, some researchers propose that ER is not a unitary construct and instead, involves multiple components (Gratz and Roemer, 2004). Specifically, vmHRV has been shown to predict ER primarily in the context of strategies (Thayer and Lane, 2000; Aldao and Mennin, 2012), that is, people with higher vmHRV are often more successful at regulating emotions when told to use a particular ER strategy. However, this work may not capture other facets of ER, such as emotional clarity and acceptance of emotions, as such facets have been shown to undermine successful ER (Gratz and Roemer, 2004) and thus, may have subsequent effects on health. Given that little is known about the relationship between vmHRV and different facets of ER, the following investigation not only attempts to link vmHRV with self-reported ER, but it also aims to understand which facets of ER are most strongly associated with vmHRV.

### Traits that relate to emotion regulation failure and lower resting heart rate variability

It is proposed that both lower vmHRV and difficulties in ER are characteristic of certain psychopathological states (Thayer and Lane, 2000). For example, research has provided compelling evidence that trait anxiety is associated with both lower vmHRV and ER capabilities. Likewise, rumination has also been associated with lower vmHRV and ER capabilities. In fact, both higher trait anxiety and rumination, and lower vmHRV and difficulties in ER are characteristic of major psychopathological disorders, such as major depressive disorder, generalized anxiety disorder, and post-traumatic stress disorder (Friedman and Thayer, 1998; Thayer and Lane, 2000; Brosschot et al., 2006; Friedman, 2007). Thus, while many variables can influence the link between inhibitory control and ER, the following investigation takes both trait rumination and anxiety into consideration, as both may serve as “third variables” in the relationship between vmHRV and ER.

### The present study

The present study aimed to explore the association of resting vmHRV and self-reported difficulties in ER. We hypothesized that those with lower resting vmHRV would report greater difficulties in everyday ER. However more specifically, the following investigation attempts to understand what day-to-day difficulties in ER resting vmHRV *best* predicts, beyond possible psychological confounds, including both trait rumination and anxiety. Overall, we sought to provide evidence of specific facets of ER individuals with low vmHRV perceive most difficulties with, while providing recommendations for future research on ER, HRV, and well-being.

## Methods and materials

### General procedure

Data were pooled across five studies conducted within the Emotions and Quantitative Psychophysiology lab. Subjects were recruited from the Research Experience Program (REP) pool at The Ohio State University, allowing students to participate in research for partial class credit in an introductory level psychology course. Funding from The Ohio State University College of Social and Behavioral Sciences and College of Arts and Sciences also allowed us to recruit and compensate participants outside of the REP pool resulting in a diverse sample across the university (i.e., students from various majors and cohorts).

No individual participated in more than one of the five studies.

We asked all participants not to smoke, undergo vigorous physical activity, or drink caffeine 6 h prior to the experiment. Each study was approved by the institutional review board, and all participants signed written informed consent. Data from the five studies have not been submitted or accepted for publication elsewhere; however, results unrelated to the current data are publically available as Theses (Cash, 2014; Williams, 2014).

In all studies, participants were placed in a soundproof experimental room, equipped with a camera and microphone for safety and instructional reasons, and a high definition TV for stimuli presentation. Participants were given a detailed explanation of the procedures that would take place without indicating the specific hypothesis under the study or manipulations applied. Electrocardiogram (ECG) leads were attached to the subjects and while in a separate control room, the experimenter led the subjects to the initial phases of the experiment. All participants first completed a 5-min baseline-resting period, where participants, while spontaneously breathing, sat and viewed a blank, gray screen, and were instructed not to move or fall asleep. Participants either completed an experimental task^1^ followed by a set of self-report questionnaires, or completed a set of self-report questionnaires followed by an experimental task. The total duration for each study was approximately 60 min.

### Heart rate variability

Cardiac activity data was recorded continuously throughout each experiment via a 3-lead ECG at a 1000 Hz sampling rate using a Mindware™ 2000D (MW2000D) Impedance Cardiograph package. Resting vmHRV was assessed during a 5-min baseline (spontaneous breathing and resting state) period prior to any experimental task. Electrodes were placed (1) below the right clavicle, (2) on the left side of the abdomen (below the heart), and (3) on the right side of the abdomen. The variability between successive R-spikes (or variability within inter-beat-intervals, IBIs) was obtained from ECG recordings to calculate HRV. Participants' successive IBIs, in milliseconds, were extracted using HRV 2.51 Analysis software. IBIs were written in a text file and analyzed using Kubios HRV analysis package 2.0 (Tarvainen et al., 2014), allowing for the calculation of time- and frequency-domain indices of vmHRV. Artifacts within the R-to-R series were visually detected, and we applied an artifact correction level that would differentiate and remove artifacts (differing abnormal IBIs from the mean IBI) using a piecewise cubic spline interpolation method. The root mean square of successive differences (RMSSD), measured in milliseconds, was calculated and is considered to be a stable (Li et al., 2009), and valid (Thayer and Sternberg, 2010), time-domain measure of vmHRV. Studies have shown that in a spontaneous breathing and resting state, RMSSD has a trait specificity of 73% (Bertsch et al., 2012), suggesting that this one-time physiological assessment of RMSSD, particularly in a spontaneous breathing and resting state, primarily reflects trait influence (three times the state specificity) and thus, it is acceptable to construe resting RMSSD to be a physiological trait measure. PNN50 was also calculated, which is defined as the percentage of R-R intervals that differed by greater than 50 ms. Autoregressive estimates were also calculated, yielding high frequency power HRV (HF-HRV, 0.15–0.4 Hz) (Thayer and Sternberg, 2010). Whereas some have suggested that under certain circumstances RMSSD may reflect sympathetic influences (Berntson et al., 2005), in the present study RMSSD correlated highly with HF power (r = 0.90, *p < 0.001*), suggesting that RMSSD is primarily vagally-mediated and as such, we report vmHRV results using RMSSD. Results were identical using HF-HRV and PNN50 (results not shown).

Additionally, high-frequency peak values (HF peak) were obtained from the autoregressive analysis as a measure of respiration frequency to control for potential bias (Thayer et al., 2002). RMSSD values were natural log transformed (ln) to fit assumptions of linear analyses (Ellis et al., 2008). Mean (3.85) and standard deviation (0.49) values for RMSSD in the current study are comparable to average values (3.49) and standard deviations (0.26) reported elsewhere (Nunan et al., 2010).

### Self-report questionnaires

Perceived difficulties in ER were assessed using the *Difficulties in Emotion Regulation Scale* (DERS). The DERS is comprised of 36-items and six sub-scales designed to measure different facets of difficulties in ER (Gratz and Roemer, 2004). Participants are asked to respond on a scale from 1 (*almost never*) to 5 (*almost always*) (example item: “*When I'm upset, I acknowledge my emotions*”). Its subscales include (i) *non-acceptance of emotional responses* (NONACC; Cronbach's *α* = 0.87); (ii) *difficulties engaging in goal-oriented behavior when experience negative emotions* (GOALS; *α* = 0.84); (iii) *difficulties in controlling impulsive behavior when experiencing negative emotions* (IMPULSE; *α* = 0.80); (iv) *lack of emotional awareness* (AWARE; *α* = 0.73); (v) *lack of strategies to regulate emotions* (STRAT; *α* = 0.79); (vi) *lack of emotional clarity* (CLARITY; *α* = 0.79). The DERS showed excellent internal-consistency in the current investigation (*α* = 0.88).

Rumination was assessed using the 22-item Ruminative Responses Scale (RRS; Conway et al., 2000). Participants answered on a scale from 1 (*almost never*) to 4 (*almost always*), (sample item: *How often do you think* “*What am I doing to deserve this?*”,) with higher numbers representing greater trait rumination. The RRS contains three subscales, including *brooding rumination* (*wallowing and sulking*), *dampening rumination* (*sadness and despair*), and *reflective rumination* (*problem solving and analyzing*). In the present analysis, only total RRS scores were used, and this scale showed excellent internal consistency (Cronbach's *α* = 0.91).

Trait anxiety was assessed using the 20-item Spielberger Trait Anxiety Inventory (STAI-T; Spielberger, 1983). Participants answered on a scale from 1 (*almost never*) to 4 (*almost always*), (sample item: “*I feel pleasant*”.) The STAI-T showed excellent internal consistency (Cronbach's α = 0.91). Finally, height and weight were recorded and body mass index (BMI)– a measure that adjusts body weight for height–was calculated.

### Statistical analysis

All statistical tests were conducted using SPSS (ver. 19, IBM Chicago, IL, USA). To examine potential bias by pooling data across studies, two univariate analysis of variance (ANOVA) tests were conducted to examine differences in RMSSD and DERS scores across studies. Results showed that there were significant differences in mean DERS scores across studies [*F*_(4, 178)_ = 2.54, *r* = 0.221 *p < 0.041*] and marginal differences in lnRMSSD [*F*_(4, 178)_ = 2.31, *r* = 0.232 *p* = 0.060]. Thus, the five studies were given dummy codes (1–5) that were statistically controlled for in all analyses. All analyses contained additional covariates including gender (coded as 1 = male, 2 = female), ethnicity (coded as 1 = European American, 2 = Other), trait anxiety (STAI-T scores), trait rumination (RRS scores), respiration (as indexed by HFpeak values), age (in years), and BMI (Kg/M^2^), all of which have been shown to be related to vmHRV (Dishman et al., 2000; Brosschot et al., 2006; Hu, 2011; Thayer et al., 2011; Koenig et al., 2014; Hill et al., 2015).

Multiple hierarchical regression tests were conducted to examine the relationship between lnRMSSD as a continuous variable and DERS (and subscales) scores controlling for aforementioned covariates. Step one included BMI, age, gender, ethnicity, and experiment as predictors. Step 2 added STAI-T and RRS scores, and step 3 added lnRMSSD and HF peak values as predictors of DERS total and subscale scores. Partial correlation coefficients and associated significance levels between lnRMSSD, covariates, and DERS (and subscales) scores are reported. In addition, a median split was performed on log-transformed RMSSD (RMSSD median value: 46.4586; lnRMSSD median value = 3.8386) to stratify subjects into groups with high and low resting vmHRV. Independent samples *t*-tests were conducted to explore potential differences between groups on all included variables. All tests were two-tailed and were analyzed using a set level of significance of *p > 0.05*.

## Results

**Figure 1:**
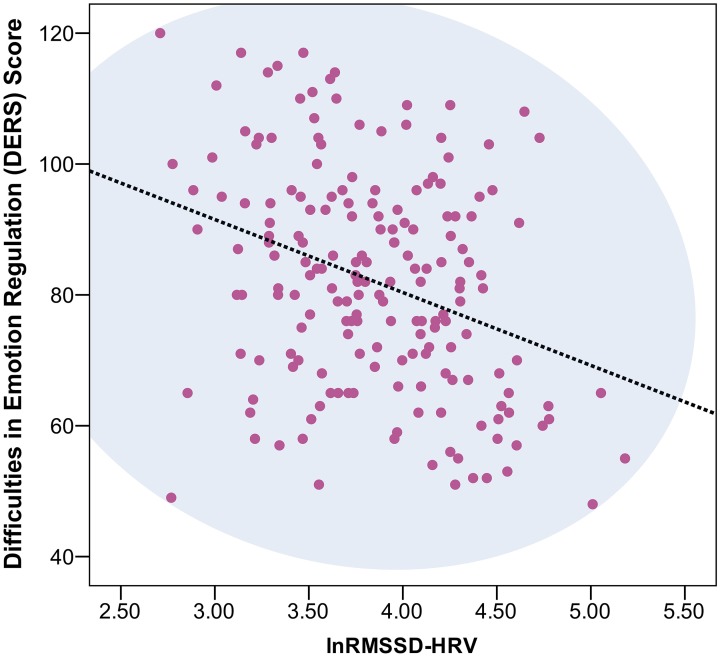
**Scatterplot of vmHRV and Difficulties in Emotion Regulation Scale (DERS) scores**. This figure represents a scatterplot between RMSSD (in milliseconds and natural log (ln) transformed and difficulties in emotion regulation scale (DERS) total scale scores (*r* = −0.325, *p < 0.001*).

Across the five experiments, 199 participants were enrolled. Nine individuals were removed due to missing data. Seven individuals yielded DERS scores ± 2 standard deviations from the mean and were removed from the overall analyses, leaving data from a total of 183 undergraduate students (98 female, 60 minority^2^, age range: 18–35, mean age = 19.34, standard deviation = 2.18) available for analyses. No outliers were removed due to abnormal lnRMSSD values (normal distribution). Independent sample *t*-tests showed that the low lnRMSSD group had higher STAI-T, RRS, DERS, and DERS subscale scores (each *p < 0.05*, with the exception of AWARE; see Table 1). Correlation results showed a significant negative relationship between lnRMSSD and RRS (r = −0.180, *p < 0.05*), STAI-T (r = −0.261, *p < 0.001*), and, as predicted, DERS total (r = −0.325, *p < 0.001*) scores (Figure 1), in addition to all DERS subscales with the exception of the AWARE subscale –Table 2 contains a correlation matrix of all psychological variables and lnRMSSD.

**Table 1:** **VmHRV group comparisons on all variables**.

	**Range of Data (min, max)**	**High vmHRV**	**Low vmHRV**	**Effect Sze (*r*)**	***p***
*n*		91	92		
Age	18, 35	19.38 (1.97)	19.29 (2.38)	0.010	*0.778*
BMI	16.54, 47.51	24.30 (4.99)	23.64 (4.51)	0.070	*0.348*
RMSSD	15.01, 178.02	73.57 (24.04)	32.33 (7.97)	0.757	***0.000***
lnRMSSD	2.71, 5.18	4.26 (0.28)	3.44 (0.27)	0.829	***0.000***
HF Peak	0.16, 0.40	0.266 (0.047)	0.275 (0.046)	0.100	*0.183*
DERS	48, 120	77.84 (16.74)	86.27 (16.00)	0.250	***0.001***
NONACC	6, 29	12.09 (4.89)	13.85 (5.53)	0.167	***0.024***
GOALS	5, 25	12.96 (4.19)	14.72 (4.34)	0.202	***0.006***
IMPULSE	6, 25	9.59 (3.30)	11.05 (4.26)	0.189	***0.01***
AWARE	7, 30	16.91 (4.61)	17.15 (4.57)	0.031	*0.724*
STRAT	8, 31	15.41 (4.78)	17.33 (4.78)	0.184	***0.012***
CLARITY	5, 24	12.17 (3.76)	10.88 (3.76)	0.145	***0.021***
STAIT	23, 69	39.10 (9.84)	42.72 (9.12)	0.187	***0.011***
RRS	22, 73	41.03 (11.34)	45.30 (11.55)	0.184	***0.011***

*This table shows the range of data and mean (standard deviation in brackets) values on baseline measures between high and low vmHRV groups, effect sizes (r) for each test, and p-values on the difference between groups (p-values italicized; bolded p-values, p < 0.05). HF peak, index of respiration; RMSSD, root mean of the squared successive differences—index of vmHRV; DERS, difficulties in regulation scale; RRS, ruminative responses scale; STAI-T, trait anxiety; NONACC, non-acceptance subscale; GOALS, goals subscale; IMPULSE, impulse control subscale; AWARE, emotional awareness subscale; STRAT, emotional regulation strategies subscale; CLARITY, emotional clarity subscale. It is important to note, only two participants were older than the age of 25*.

**Table 2:** **Correlation matrix of HRV and psychological variables**.

	**lnRMSSD**	**RRS**	**STAI-T**	**DERS**	**GOALS**	**IMPULSE**	**AWARE**	**STRAT**	**CLARITY**	**NONACC**
lnRMSSD	–									
RRS	−0.180^*^	–								
STAI-T	−0.261^***^	0.688^***^	–							
DERS Total	−0.325^***^	0.580^***^	0.688^***^	–						
GOALS	−0.219^**^	0.416^***^	0.461^***^	0.668^***^	–					
IMPULSE	−0.244^***^	0.386^***^	0.459^***^	0.678^***^	0.415^***^	–				
AWARE	−0.117	−0.111	0.099	0.281^**^	−0.090	0.035	–			
STRAT	−0.241^**^	0.635^***^	0.698^***^	0.818^***^	0.580^***^	0.605^***^	**−**0.016	–		
CLARITY	−0.242^**^	0.238^**^	0.370^***^	0.589^***^	0.162^*^	0.227^**^	0.336^***^	0.272^***^	–	
NONACC	−0.167^*^	0.528^***^	0.441^***^	0.675^***^	0.396^***^	0.303^***^	−0.146^*^	0.525^***^	0.300^***^	–

This table shows zero-order correlation coefficients (statistically significant coefficients bolded) between all variables. HF peak, index of respiration; RMSSD, root mean of the squared successive differences—index of vagally-mediated HRV, RRS: ruminative responses scale; STAI-T, trait anxiety; DERS, difficulties in regulation scale; NONACC, non acceptance subscale; GOALS, goals subscale; IMPULSE, impulse control subscale; AWARE, emotional awareness subscale; STRAT, emotional regulation strategies subscale; CLARITY, emotional clarity subscale.

* p < 0.05

** p < 0.01

*** *p < 0.001*.

Multiple hierarchical regression models showed that, after controlling for experiment type, age, ethnicity, gender, BMI, STAI-T scores, RRS scores, and HF peak (respiration) values, lnRMSSD was significantly associated with DERS total scores (r_partial_ = −0.222, *p < 0.01*). Moreover, lnRMSSD was significantly associated with scores obtained from the CLARITY (r_partial_ = −0.175, *p < 0.05*) and IMPULSE (r_partial_ = −0.155, *p < 0.05*) subscales (see Table 3 for partial correlations between predictors and all DERS variables, see Table 4 for a summary of regression models).

**Table 3:** **Partial correlations between predictors and DERS Scores**.

	**DERS**	**NONACC**	**GOALS**	**IMPULSE**	**AWARE**	**STRAT**	**CLARITY**
LnRMSSD	−0.222^**^	−0.036	−0.113	−0.155^*^	−0.133	−0.088	−0.175^*^
HF peak	−0.088	0.065	0.014	−0.007	−0.158^*^	−0.104	−0.082
BMI	−0.069	0.018	−0.111	−0.054	−0.031	−0.088	0.051
Ethnicity	−0.149^*^	−0.158^*^	−0.002	−0.053	−0.042	0.029	−0.215^**^
Gender	0.007	0.081	0.165^*^	−0.048	−0.253^***^	0.111	−0.011
Age	−0.204^**^	−0.038	−0.100	−0.153^*^	−0.153^*^	−0.020	−0.170^*^
STAI-T	0.469^***^	0.122	0.237^**^	0.246^***^	0.225^**^	0.456^***^	0.273^***^
RRS	0.212^***^	0.351^***^	0.157^*^	0.135	−0.262^***^	0.299^***^	−0.033
Experiment	0.078	−0.067	0.046	0.106	0.126	−0.025	0.061

This table shows partial correlation coefficients (statistically significant coefficients bolded) between predictor variables and DERS total and subscale scored, yielded from the hierarchical regression model. Specifically, these partial correlation coefficients show the relationship between predictor variables (e.g., lnRMSSD) and DERS and subscale scores while partialing out the influence of the other predictor variables (e.g., HF peak, BMI, Ethnicity, Gender, Age, STAI-T, RRS, and experiment). HF peak, index of respiration; RMSSD: root mean of the squared successive differences—index of vagally-mediated HRV DERS, difficulties in regulation scale; RRS, ruminative responses scale; STAI-T, trait anxiety; NONACC, non acceptance subscale; GOALS, goals subscale; IMPULSE, impulse control subscale; AWARE, emotional awareness subscale; STRAT, emotional regulation strategies subscale; CLARITY, emotional clarity subscale.

* p < 0.05

** p < 0.01

*** *p < 0.001*.

**Table 4:** **Summary of hierarchical regression analysis for variables predicting DERS Scores**.

**Step**			**DERS total**			**IMPULSE**			**CLARITY**		
	**1**	**2**	**3**	**1**	**2**	**3**	**1**	**2**	**3**
Age	−0.273^***^	−0.137^*^	−0.148^***^	−0.221^***^	−0.133^**^	−0.142^***^	−0.224^***^	−0.152^*^	−0.163^*^
Ethnicity	−0.032	−0.108^*^	−0.106^**^	−0.001	−0.050	−0.048^*^	−0.164^*^	−0.207^**^	−0.205^**^
Gender	−0.005	0.000	0.004^*^	−0.050	−0.047	−0.043	−0.020	−0.015	−0.010
Experiment	0.053	0.037	0.056	0.086	0.075	0.098	0.050	0.040	0.058
BMI	−0.032	−0.069	−0.050	−0.040	−0.064	−0.050	0.045	0.028	0.049
STAI-T		0.541^***^	0.507^***^		0.351^***^	0.312^***^		0.393^***^	0.359^***^
RRS		0.202^**^	0.119^**^		0.135	0.135		−0.037	−0.040
HF peak			−0.060			−0.006			−0.074
lnRMSSD			−0.161^**^			−0.143^*^			−0.167^*^
Constant	125.32	59.70	88.48	18.66	8.821	13.74	19.41	11.81	18.78
R2	0.075	0.535	0.559	0.049	0.246	0.264	0.069	0.198	0.226

This table shows standardized beta (β) coefficients with associated significance levels at each step in the regression model. HF peak, index of respiration; RMSSD, root mean of the squared successive differences—index of vmHRV DERS, difficulties in regulation scale; RRS, ruminative responses scale; STAI-T, trait anxiety; IMPULSE, impulse control subscale; CLARITY, emotional clarity subscale.

* p < 0.05

** p < 0.01

*** *p < 0.001*.

## Discussion

The present study is the first study to report a significant negative association between resting vmHRV and self-reported difficulties in everyday ER, as indexed by the DERS. Both general anxiety and ruminative tendencies were correlated with vmHRV and difficulties in ER in the current investigation, such that greater anxiety and rumination were associated with lower vmHRV and greater difficulties in ER. However, after controlling for these psychological covariates, our results provide evidence that those with lower resting vmHRV have greater difficulties with everyday ER.

The DERS scale examines six different facets of ER difficulties. Thus, we were able to examine particular facets of ER and their relation to vmHRV. We found that those with lower vmHRV report greater difficulties with emotional clarity and emotional-impulse control. A lack of emotional clarity can be defined as lacking clarity or understanding of conscious or unconscious negative emotions (Gratz and Roemer, 2004). Thus, individuals with lower vmHRV may find it difficult to *identify* prepotent emotional responses and thus, are unable to inhibit and/or regulate them adaptively and consistently.

Additionally, we found vmHRV to be associated with difficulties in controlling impulsive behavior. Difficulty in impulse control is defined as having difficulties in controlling impulsive behaviors when experiencing negative emotions (Gratz and Roemer, 2004). Insomuch that individuals with low vmHRV show lesser inhibitory control in comparison to their counterparts that exhibit higher vmHRV, this relationship corresponds to prior theory—hypothesizing that those with lower vmHRV are unable to *inhibit* impulsive behaviors associated with negative emotions.

In addition, it is important to note that high and low HRV group analyses revealed the strongest difference between groups on the GOALS subscale of the DERS in comparison to the other subscales. The GOALS subscale represents difficulties in engaging in goal-oriented behavior—actions that are in accordance with present goals and motivations—when negative emotions are present. Therefore, our results suggest that at the group level, those with low HRV likely experience more difficulties in concentrating and accomplishing goal-oriented tasks when experiencing negative emotions in comparison to individuals with high HRV. These results are in line with previous work, suggesting a link between lower resting HRV and greater difficulties in both goal attainment and goal commitment (Verkuil et al., 2010).

Past research has examined the link between vmHRV and ER abilities using specific ER tasks and ER strategies (Melzig et al., 2009; Aldao and Mennin, 2012). The present study adds to this literature by showing that resting vmHRV is associated with a self-report measure of *everyday perceived* difficulties in ER, and strongest with difficulties in emotional clarity, impulse control, and in some cases, goal-oriented behavior. One study found that those with low ER capabilities, as indexed by the DERS, did not show physiological recovery, as indexed by phasic changes in vmHRV, following a film-elicited emotion procedure (Berna et al., 2014). Our data are consistent with, but also extend these findings, suggesting that individuals with lower baseline-resting vmHRV experience difficulties/failures with everyday ER, and are able to report such difficulties.

### Limitations and future directions

One limitation of the current investigation is that the sample consisted of college students and thus, the current results may not extend to other age ranges. While we are confident that difficulties in ER will be related to vmHRV in all age groups, we are not sure that difficulties in impulse control and emotional clarity will be closely associated with vmHRV as in the current sample. Thus, future research should attempt to examine the DERS and vmHRV relationship in individuals of various age ranges.

A second limitation of the current investigation is that, while we controlled for anxiety and rumination, other possible psychological covariates, such as perceived stress and depressive symptoms, were not statistically controlled for in the current analysis. Thus, future research should ensure that the current results remain strong and significant when considering other possible psychological covariates.

Moreover, while the relationship between resting vmHRV and difficulties in ER is strong, it is not perfect. Indeed, this could be due to the possible mediating role that rumination and/or anxiety plays on the link between vmHRV and difficulties in ER, especially given the modest correlation between these variables. Nevertheless, within the current sample, there are individuals with adequate inhibitory abilities, as indexed by higher vmHRV, who perceive difficulties in ER. Conversely, there are individuals with lower vmHRV who do not perceive difficulties in ER, despite their lack of inhibitory abilities. This suggests a third variable at play—which could be as simple as the number of emotional encounters (lack of ER experiences) or as complex as individuals' emotional numbness, intelligence, or regulatory skills (lack of ER practice). In fact, recent research suggests that ER should be seen as a motivational process, such that the motivation to engage in ER, in addition to employing ER strategies, differs from person to person, independent of their actual ability (Aldao and Mennin, 2014). Thus, although an individual is equipped to deal with negative emotions (i.e., have high resting vmHRV), they may be unmotivated (or motivated differently) to regulate those emotions and as such, perceives more difficulties. Future researchers should attempt to identify these individuals, in addition to the variable accounting for the discrepancy between resting vmHRV and perceived difficulties in ER.

Finally, the current investigation is cross-sectional by nature and thus, causation cannot be determined in the present study. However according to the Model of Neurovisceral Integration, it is likely that vmHRV is influencing ER failure and success. On the other hand, other research proposes that maladaptive/adaptive ER decreases/increases acute vmHRV. Thus, it is also likely that those who are maladaptive in their emotional responding have subsequent maladaptive chronic physiological responses. Future research is warranted to explore such notions.

## Conclusions

The present investigation shows that resting vmHRV is related to individuals' everyday perceptions of difficulties to regulate their emotions, especially difficulties with emotional clarity and impulse control. Thus, in order to increase adaptive ER success in those with lower vmHRV, these data suggest that individuals should: (1) work to increase vmHRV, as doing so may increase ER; (2) work to understand and identify negative emotions, so that they have the opportunity to adaptively regulate such distressing emotions and; (3) these individuals should work to inhibit impulsive behavior, as these responses may be undesirable. The current study both supports the NIM and extends prior research on ER and vmHRV, giving two facets of ER (impulse control and emotional clarity) that should be of particular focus when investigating the link between resting vmHRV and ER. Overall, the current data support the notion that vmHRV serves as a proxy of ER ability, especially when negative emotions are unclear and resulting behavior is impulsive. We live in a world where we often experience both simple and complex emotions—we hope that future research gives special attention to these facets of ER as doing so may assist in understanding the link between ER and negative physical and mental health outcomes and longevity.

### Conflict of interest statement

The authors declare that the research was conducted in the absence of any commercial or financial relationships that could be construed as a potential conflict of interest.

## Footnotes

^1^Experimental tasks in each study were specific to the primary aims of each investigation. Presumably, these tasks did not alter influence the current results.

^2^There were a total of 17 Asian Americans, 28 African Americans, 1 Brazilian, 12 Hispanic Americans, 2 Native Americans.
